# 精准治疗时代下广泛期小细胞肺癌联合治疗研究进展

**DOI:** 10.3779/j.issn.1009-3419.2026.101.14

**Published:** 2026-07-20

**Authors:** Hanrui CHEN, Liwen XU, Yixuan CHENG, Lei ZHOU

**Affiliations:** 200032 上海，上海中医药大学附属龙华医学院; Longhua Hospital Affiliated to Shanghai University of Traditional Chinese Medicine, Shanghai 200032, China

**Keywords:** 肺肿瘤, 小细胞肺癌, 免疫疗法, 免疫检查点抑制剂, 抗血管生成, 靶向治疗, 放疗, 中医药, 精准医学, Lung neoplasms, Small cell lung cancer, Immunotherapy, Immune checkpoint inhibitors, Angiogenesis inhibitors, Targeted therapy, Radiotherapy, Traditional Chinese medicine, Precision medicine

## Abstract

广泛期小细胞肺癌（extensive-stage small cell lung cancer, ES-SCLC）进展迅速、复发率高、预后较差，仍是肺癌治疗领域的难点。近年来，免疫检查点抑制剂联合依托泊苷含铂化疗成为ES-SCLC的一线治疗标准，推动ES-SCLC由传统化疗向多模式联合及精准分层转变，为疗效突破带来新的机遇和挑战。本文基于临床治疗路径，从标准化方案、维持策略、临床局限性及前沿研究热点等方面，综述了ES-SCLC的化疗、免疫、靶向、放疗、中医药等多学科联合治疗研究进展，以期在精准治疗时代为ES-SCLC的个体化治疗与全程管理提供参考。

## Abstract (en)

Extensive-stage small cell lung cancer (ES-SCLC) is characterized by rapid progression, a high risk of recurrence, and poor prognosis, and remains a major therapeutic challenge in lung cancer. In recent years, immune checkpoint inhibitors combined with Platinum-Etoposide chemotherapy have become the standard first-line treatment for ES-SCLC, driving a shift from conventional chemotherapy toward multimodal combination strategies and precision-guided patient stratification, while creating both new opportunities and challenges for further therapeutic improvement. Following the clinical treatment pathway, this review summarizes advances in multidisciplinary combination treatment for ES-SCLC, including chemotherapy, immunotherapy, targeted therapy, radiotherapy, and traditional Chinese medicine, with emphasis on standard regimens, maintenance therapy, clinical limitations, and emerging research directions. This review aimed to provide a reference for individualized treatment and comprehensive disease management of ES-SCLC in the era of precision medicine.

根据GLOBOCAN2026数据^[1]^，肺癌仍是全球肿瘤相关死亡的首位原因之一。小细胞肺癌（small cell lung cancer, SCLC）占肺癌的13%-15%，约70%患者在初诊时已处于广泛期，5年生存率不足10%^[2]^。免疫检查点抑制剂改善了广泛期SCLC（extensive-stage SCLC, ES-SCLC）的生存结局，但复发和耐药仍普遍存在。^随着分子分型研究深入，^SCLC的分子异质性及不同亚型特征逐渐受到重视^[3]^。

本文围绕ES-SCLC的临床治疗路径，综述一线化免治疗及强化策略、传统分子通路靶向治疗的局限、新型肿瘤表面抗原靶向治疗、局部放疗与全身治疗的整合，以及中医药在综合治疗中的辅助作用，并探讨精准治疗时代下ES-SCLC面临的主要挑战。

## 1 ES-SCLC化免治疗的标准模式、强化策略与探索

### 1.1 依托泊苷+含铂化疗联合程序性细胞死亡受体1（programmed cell death receptor 1, PD-1）/程序性细胞死亡配体1（programmed cell death-ligand 1, PD-L1）抑制剂：一线治疗标准确立

依托泊苷联合含铂化疗是ES-SCLC的基础治疗方案，主要包括依托泊苷联合顺铂（Etoposide plus Cisplatin, EP）和依托泊苷联合卡铂（Etoposide plus Carboplatin, EC），初始客观缓解率（objective response rate, ORR）可达60%-80%，但多数患者在4-6个月内进展，后线化疗获益有限^[4,5]^。PD-1及其配体PD-L1是肿瘤免疫逃逸的重要调控通路^[6]^。以IMpower133^[7]^、CASPIAN^[8]^为代表的国际III期研究率先证实，PD-L1抑制剂阿替利珠单抗或度伐利尤单抗联合EC/EP方案可显著改善无进展生存期（progression-free survival, PFS）和总生存期（overall survival, OS）。随后，CAPSTONE-1^[9]^、ASTRUM-005^[10]^、EXTENTORCH^[11]^、RATIONALE-312^[12]^等多项中国原创III期临床研究相继取得阳性结果，并推动阿得贝利单抗、斯鲁利单抗、特瑞普利单抗、替雷利珠单抗及索卡佐利单抗^[13]^成为一线治疗的选择。

但并非所有化免联合研究均取得阳性结果。KEYNOTE-604研究^[14]^发现帕博利珠单抗联合EP方案虽显著改善PFS，但未显著延长OS，可能与治疗组基线脑转移患者比例较高（14.5% vs 9.8%）有关。总体而言，PD-1/PD-L1抑制剂联合含铂化疗使ES-SCLC患者OS由约10个月延长至12-15.8个月，5年生存率提高至10%-12%^[15]^，成为目前ES-SCLC的一线标准治疗方案（表1）。临床实际应用需综合考虑患者体能状态、基础疾病、药物可及性、医保覆盖及经济负担等因素。个体化治疗策略仍是当前ES-SCLC临床管理的重要原则。

**表1:** ES-SCLC一线治疗的III期临床试验结果

Target	Trial	Treatment arms	mPFS (mon)	HR (95%CI), P	mOS (mon)	HR (95%CI), P
PD-L1	IMpower133	Atezolizumab+EC vs Placebo+EC	5.2 vs 4.3	0.77 (0.62-0.96) P=0.02	12.3 vs 10.3	0.76 (0.60-0.95) P=0.0154
	CASPIAN	Durvalumab+EP/EC vs EP/EC alone	5.1 vs 5.4	0.78 (0.66-0.96) P=0.0032	12.9 vs 10.3	0.73 (0.59-0.91) P=0.0047
	CAPSTONE-1	Adebrelimab+EC vs Placebo+EC	5.8 vs 5.6	0.67 (0.54-0.83) P<0.001	15.3 vs 12.8	0.72 (0.58-0.90) P=0.0017
	NCT04878016	Socazolimab+EC vs Placebo+EC	5.5 vs 4.4	0.569 (0.46-0.71) P<0.001	13.9 vs 11.6	0.799 (0.65-0.98) P=0.0158
PD-1	ASTRUM-005	Serplulimab+EC vs Placebo+EC	5.7 vs 4.3	0.48 (0.38-0.59) P: NR	15.4 vs 10.9	0.63 (0.49-0.82) P<0.001
	EXTENTORCH	Toripalimab+EP/EC vs Placebo+EP/EC	5.8 vs 5.6	0.67 (0.54-0.82) P=0.0002	14.6 vs 13.3	0.798 (0.65-0.98) P=0.033
	RATIONALE-312	Tislelizumab+EP/EC vs Placebo+EP/EC	4.7 vs 4.3	0.64 (0.52-0.78) P<0.001	15.5 vs 13.5	0.75 (0.61-0.93) P=0.004
	KEYNOTE-604	Pembrolizumab+EP vs Placebo+EP	4.5 vs 4.3	0.75 (0.61-0.91) P=0.0023	10.8 vs 9.7	0.80 (0.64-0.98) P=0.016

ES-SCLC: extensive-stage small cell lung cancer; CI: confidence interval; EC: Etoposide plus Carboplatin; EP: Etoposide plus Cisplatin; HR: hazard ratio; mOS: median overall survival; mPFS: median progression-free survival; PD-1: programmed cell death receptor 1; PD-L1: programmed cell death-ligand 1; NR: not reported.

### 1.2 化免诱导后的维持治疗：标准免疫维持与强化策略探索

对于化免诱导后疾病未进展者，免疫维持已成为常用模式。多项III期研究均采用4-6个周期诱导后免疫维持，并获得持续生存获益。IMpower133探索性分析结果^[16]^显示，初始治疗达到完全缓解（complete response，CR）或部分缓解（partial response，PR）的患者从维持治疗中获益更为显著。在此基础上，强化维持策略正受到关注。IMforte III期临床研究^[17]^显示，与阿替利珠单抗单药维持相比，芦比替丁联合阿替利珠单抗可显著延长PFS（5.4 vs 2.1个月）和OS（13.2 vs 10.6个月），但血液学毒性增加，3级以上治疗相关不良事件（treatment-related adverse events, TRAEs）发生率达38%，5级TRAEs发生率为5%，临床应用需权衡获益与安全性。

### 1.3 抗血管生成联合化免治疗：一线强化方案的突破与局限

血管生成药物可通过改善肿瘤血管及免疫微环境，增强化疗和免疫治疗效应^[18]^。ETER701研究^[19]^显示，贝莫苏拜单抗联合安罗替尼及EC方案为目前ES-SCLC一线III期研究中最长的中位OS。但该方案因TRAEs导致治疗中断的比例较高，临床应用仍需结合体能状态、器官功能及不良反应管理能力综合评估。CeLEBrATE^[20]^、BEAT-SC研究^[21]^显示，贝伐珠单抗联合阿替利珠单抗及化疗虽可改善PFS，但尚未显示明确OS获益，可能与随访时间尚短有关。抗血管生成联合化免治疗具有一定前景，但不同药物组合的临床获益并不一致（表2），仍需进一步明确优势人群及安全性。

**表2:** 抗血管生成药物联合标准化免治疗在ES-SCLC中的临床研究结果

Targets	Treatment line and phase	Trial	Treatment arms	mPFS (mon), HR (95%CI), P	mOS (mon), HR (95%CI), P
VEGF/VEGFR+ PD-1/PD-L1	First line, phase III	ETER701	Anlotinib+Benmelstobart+EC vs Anlotinib+EC	6.9 vs 4.2 0.32 (0.26-0.41) P<0.0001	19.3 vs 11.9 0.61 (0.47-0.79) P=0.0002
	First line, phase II	CeLEBrATE	Bevacizumab+Atezolizumab+ EP/EC	6.2 (5.4-6.6) HR/P: NR	12.9 (11.6-17.5) HR/P: NR
	First line, phase III	BEAT-SC	Bevacizumab+Atezolizumab+ EP/EC vs Placebo+Atezolizumab+EP/EC	5.7 vs 4.4 0.70 (0.54-0.90) P=0.006	13.3 vs 16.6 1.22 (0.89-1.67) P=0.2212

VEGF: vascular endothelial growth factor; VEGFR: VEGF receptor.

### 1.4 双重免疫检查点阻断：III期研究未证实生存获益

双重免疫检查点抑制剂联合治疗主要包括PD-1/PD-L1抑制剂联合细胞毒性T淋巴细胞相关抗原4（cytotoxic T lymphocyte-associated antigen-4, CTLA-4）抑制剂或^T细胞免疫球蛋白和ITIM结构域^（^T cell immunoglobulin and ITIM domain, TIGIT^）抑制剂，旨在协同解除免疫抑制^[22]^。CheckMate 032研究^[23]^显示，纳武利尤单抗联合伊匹木单抗策略对后线SCLC可提高ORR（21.9% vs 11.6%），但后续CheckMate 451^[24]^和CASPIAN^[25]^两项III期研究均未证实该策略可进一步改善PFS或OS（表3）。TIGIT抑制剂联合PD-1/PD-L1抑制剂及化疗在早期研究中亦显示一定疗效，但毒性有所增加^[26]^。III期SKYSCRAPER-02研究^[27]^未能证实在阿替利珠单抗联合EC方案基础上加用TIGIT抑制剂可进一步改善PFS或OS（表3）。双重免疫检查点抑制虽具有理论基础，但迄今III期研究未证实其可改善ES-SCLC生存，且可能增加治疗毒性。

**表3:** 双重免疫检查点抑制剂在ES-SCLC中的临床研究结果

Targets	Treatment line and phase	Trial	Treatment arms	mPFS (mon) (95%CI)	mOS (mon) (95%CI)
PD-L1+ CTLA4	First line, phase III	CASPIAN	Durvalumab+Tremelimumab+EP EP alone	4.9 (4.7-5.9) 5.4 (4.8-6.2)	10.4 (9.6-12.0) 10.5 (9.3-11.2)
PD-1+ CTLA4	Second line, phase I/II	CheckMate 032	Nivolumab+Ipilimumab Nivolumab	1.4 (1.3-1.4) 1.5 (1.4-2.2)	5.7 (3.8-7.6) 4.7 (3.1-8.3)
	First-line maintenance, phase III	CheckMate 451	Nivolumab+Ipilimumab Nivolumab Placebo	1.7 (1.5-2.6) 1.9 (1.6-2.6) 1.4 (1.4-1.5)	9.2 (8.2-10.2) 10.4 (9.5-12.1) 9.6 (8.2-11.0)
PD-1+ TIGIT	First line, phase I	AdvanTIG-105	Ociperlimab+Tislelizumab+ Platinum	4.9 (4.2-5.7)	NR
PD-L1+ TIGIT	First line, phase III	SKYSCRAPER-02	Tiragolumab+Atezolizumab+EC Placebo+Atezolizumab+EC	5.1 vs 5.4 1.08 (0.89-1.38)	12.8 vs 12.9 1.09 (0.88-1.35)

CTLA-4: cytotoxic T-lymphocyte-associated protein 4; TIGIT: T cell immunoglobulin and ITIM domain.

## 2 ES-SCLC传统通路靶向治疗的有限获益与转化困境

与非小细胞肺癌（non-small cell lung cancer, NSCLC）中表皮生长因子受体（epidermal growth factor receptor, EGFR）、间变性淋巴瘤激酶（anaplastic lymphoma kinase, ALK）等可操作性驱动基因不同，SCLC长期缺乏成熟的驱动基因靶向治疗体系^[28]^。聚二磷酸腺苷核糖聚合酶（poly(ADP-ribose) polymerase, PARP）抑制剂及Aurora激酶A（Aurora kinase A, AURKA）抑制剂虽在部分早期研究或特定分子亚组中显示潜在活性，但尚未形成明确生存获益，亦未进入常规临床应用^[29,30]^。在肿瘤血管生成及细胞代谢信号通路方面，血管内皮生长因子（vascular endothelial growth factor, VEGF）在SCLC中普遍高表达，并与肿瘤侵袭性、化疗耐药及不良预后密切相关^[31]^。安罗替尼作为多靶点酪氨酸激酶抑制剂，在ALTER-1202等研究中显示可显著延长复发SCLC患者的PFS（4.1 vs 0.7个月），并呈现OS获益趋势（7.3 vs 4.9个月），目前其单药治疗获批用于ES-SCLC三线治疗^[32]^。

传统通路靶向治疗难以取得持续疗效，主要与SCLC高度异质性及谱系可塑性有关。除TP53和RB1失活外，MYC家族扩增、BCL2高表达、PTEN缺失、Notch通路异常及NFIB扩增等改变均可参与神经内分泌分化、转移扩散、治疗敏感性及耐药演化过程^[2]^。SCLC不同分子亚型并非固定不变的遗传类别，而是受ASCL1、NEUROD1、POU2F3及炎症相关特征共同塑造的动态状态^[3]^，并可在治疗压力下发生表型转换及耐药克隆选择，从而削弱单一靶点或单一通路的持续疗效^[33]^。不同亚型在神经内分泌分化程度、免疫微环境、治疗脆弱性及潜在靶点表达方面存在差异，例如ASCL1相关亚型常伴随delta样配体3（delta-like ligand 3, DLL3）等神经内分泌相关抗原表达，而炎症型亚型可能具有更高免疫浸润水平^[34]^。未来ES-SCLC精准治疗的突破，可能不再局限于单一通路靶向药物的经验性联合，而更有赖于基于分子亚型、动态生物标志物及肿瘤表面抗原表达的综合分层与精准干预。

## 3 ES-SCLC新型肿瘤表面抗原靶向与精准治疗的破局之路

随着SCLC分子异质性及亚型特征逐步明确，基于肿瘤表面抗原的精准治疗成为研究热点。该类策略通过抗体、抗体药物偶联物（antibody-drug conjugate, ADC）或免疫细胞识别肿瘤细胞表面高表达抗原并介导定向杀伤，目前研究较为深入的靶点主要包括DLL3。DLL3是一种非典型Notch信号通路配体，是转录因子ASCL1的下游靶基因，在SCLC中高表达且具有一定的肿瘤特异性，其表达水平在化疗前后相对稳定，为靶向治疗提供了基础^[35]^。

### 3.1 靶向DLL3 T细胞衔接器（T-cell engager, TCE）：塔拉妥单抗确立后线治疗价值

DLL3靶向TCE同时结合肿瘤细胞表面的DLL3和CD3分子，激活T细胞并杀伤肿瘤细胞^[35]^。DeLLphi-301^[36]^及DeLLphi-304^[37]^研究先后证实了塔拉妥单抗在既往接受治疗的ES-SCLC患者中的抗肿瘤活性及相较传统二线化疗的生存优势（表4）。其治疗相关毒性主要为细胞因子释放综合征（cytokine release syndrome, CRS）和免疫效应细胞相关神经毒性综合征（immune effector cell-associated neurotoxicity syndrome, ICANS），其中CRS多为1-2级，经支持治疗通常可缓解。基于现有证据，塔拉妥单抗被纳入国内外指南后线治疗II级推荐。另一DLL3靶向TCE药物Obrixtamig在早期研究^[38]^中显示出一定抗肿瘤活性，其联合标准化免治疗用于一线诱导及维持阶段的安全性初步可接受，但疗效及生存获益仍需进一步验证。

**表4:** 靶向DLL3的新型药物在ES-SCLC中的临床研究结果

Targets	Treatment line and phase	Trial	Treatment arms	ORR (%), (95%CI)	DOR (mon), (95%CI)	mPFS (mon), (95%CI)	mOS (mon), (95%CI)
TCE	Third line, phase II	DeLLphi-301	Tarlatamab 10 mg Tarlatamab 100 mg	40.4 (30.7-50.7) 32.2 (22.6-43.1)	NE (6.9-NE) 8.1 (5.6-12.7)	4.3 (2.9-6.7) 3.9 (2.6-4.4)	15.2 (10.8-NE) 15.1 (10.1-NE)
	Second line, phase III	DeLLphi-304	Tarlatamab Standard second-line chemotherapy	35.0 (29.0-41.0) 20.0 (16.0-26.0)	6.9 (4.5-12.4) 5.5 (4.2-5.7)	4.2 vs 3.7 0.72 (0.59-0.88)	13.6 vs 8.3 0.60 (0.47-0.77)
ADC	Third line, phase I	NCT01901653	Rova-T	38.0 95%CI: NR	NR	4.3 (2.8-5.6)	5.8 (4.4-11.6)
	Third line, phase II	TRINITY	Rova-T	14.3 (10.1-19.4)	3.7 (2.9-4.2)	3.8 (3.2-4.1)	5.7 (4.9-6.7)
	Second line, phase III	TAHOE	Rova-T Rova-T+Topotecan	15 vs 21 95%CI: NR	3.5 (2.8-4.2) 4.9 (3.9-7.9)	3.0 vs 4.3 (1.22-1.87)	6.3 vs 8.6 (1.17-1.82)
	First-line maintenance, phase III	MERU	Rova-T Placebo	10.0 vs 5.0	4.4 (2.8-5.8) 4.7 (1.6-NE)	1.4 vs 4.0 (0.39-0.59)	8.5 vs 9.8 (0.84-1.36)

TCE: T-cell engager; ADC: antibody-drug conjugate; DLL3: delta-like ligand 3; ORR: objective response rate; DOR: duration of response; NE: not estimable; Rova-T: Rovalpituzumab tesirine; Second-line chemotherapy included Topotecan, Lurbinectedin, or Amrubicin.

### 3.2 靶向DLL3 ADC：首代药物局限与新药结构优化

ADC通过特异性抗体识别肿瘤细胞表面抗原，将细胞毒药物定向递送至肿瘤细胞实现精准杀伤^[39]^。首代DLL3靶向ADC洛伐妥珠单抗（Rovalpituzumab tesirine, Rova-T）虽在早期研究中显示一定活性，但TRINITY^[40]^、TAHOE^[41]^及MERU^[42]^等后续研究均未证实生存获益（表4），且毒性较高。其失败提示，DLL3高表达不足以单独预测ADC疗效，其临床转化除受患者选择及早期单臂研究偏倚影响外，还与ADC结构设计密切相关。Rova-T较低的药物-抗体比、PBD载荷毒性及连接子稳定性不足，可能共同限制其治疗窗^[43]^。新一代DLL3靶向ADC药物SHR-4849（IDE849）采用人源化抗DLL3 IgG1抗体、可裂解连接子及拓扑异构酶I抑制剂载荷，在早期研究^[44]^中显示出较高的抗肿瘤活性及可控的安全性，并在脑转移亚组中亦观察到较高的缓解率，但其长期生存获益仍需更高等级循证证据进一步验证。

## 4 ES-SCLC局部放疗联合全身治疗的协同策略

放疗不仅可通过诱导DNA双链断裂发挥局部抗肿瘤作用，还可促进肿瘤抗原释放、增强抗原递呈及提高肿瘤免疫原性，从而与免疫治疗产生协同效应^[45]^。随着化免联合治疗成为标准方案，放疗正由传统姑息性局部治疗逐步向联合治疗的重要组成部分转变，以期优化局部控制并延长生存。

### 4.1 胸部放疗：化免时代的应用与时序探索

CREST III期研究^[46]^显示，对于一线含铂化疗后疾病得到控制但仍存在胸内残留病灶的患者，序贯胸部放疗可提高2年OS率，并降低胸内复发风险。在化免联合治疗背景下，一项II期研究^[47]^进一步显示，阿得贝利单抗联合化疗诱导后序贯胸部放疗及免疫维持治疗可使ES-SCLC患者的中位OS达到21.4个月，为胸部放疗联合免疫治疗提供了初步循证依据。

同步胸部放疗亦受到关注。MATCH单臂II期研究^[48]^显示，阿替利珠单抗联合化疗同步低剂量胸部放疗具有较好的疗效及安全性，ORR达87.5%，并呈现较好的长期生存趋势。然而，目前放疗联合一线化免治疗的最佳时序尚无定论。同步放疗可能进一步增强免疫原性，但也可能增加肺炎等治疗相关毒性；序贯放疗安全性相对较好，其最佳应用时机仍有待随机对照研究进一步验证。

### 4.2 颅内防治与远处病灶局部控制策略

在脑转移预防方面，早期研究^[49-51]^显示，预防性脑照射（prophylactic cranial irradiation, PCI）可降低SCLC症状性脑转移发生率，并改善PFS和OS。但日本III期研究^[52]^显示，在基线脑磁共振成像（magnetic resonance imaging, MRI）阴性并接受严格MRI动态随访的条件下，PCI并未进一步改善OS，提示规律MRI监测可作为PCI之外的重要管理策略。

对于已发生脑转移的患者，全脑放疗（whole-brain radiotherapy, WBRT）仍是多发或弥漫性病灶的主要治疗方式，可改善颅内控制和神经系统症状^[53]^。对于脑转移灶数目有限、颅外病灶控制良好且能够接受密切MRI随访的患者，立体定向放射治疗（stereotactic radiosurgery, SRS）可作为选择，FIRE-SCLC多中心队列研究^[54]^显示，SRS在减少全脑放疗相关影响的同时，可获得良好的颅内控制效果。

对于寡转移或症状性病灶，局部放疗可缓解症状并改善局部控制。RTOG0937研究^[55]^提示，在PCI基础上联合胸部及转移灶巩固放疗虽可显著降低3个月疾病进展率，但未形成稳定的长期生存获益，其更适用于系统治疗有效、局部进展风险较高或存在症状负担的患者。

## 5 中医药参与ES-SCLC综合治疗的辅助价值与循证局限

中医药已广泛参与肺癌手术、放疗、化疗、靶向及免疫治疗等不同阶段，在改善症状、减轻治疗相关不良反应和提高生活质量方面具有一定临床基础^[56]^。但目前针对ES-SCLC的研究仍较少，尚缺乏统一的干预方案及明确的优势人群。

现有证据主要来自回顾性队列、小样本前瞻性研究及单中心随机对照研究。回顾性队列研究^[57]^显示，中医药联合EP方案可延长PFS（6.9 vs 4.9个月），多因素Cox分析提示中医药干预为独立保护因素。前瞻性队列研究观察“扶正解毒祛瘀法”联合化疗及PD-L1抑制剂可改善PFS（6.9 vs 5.5个月）及OS，但OS获益幅度有限（12.5 vs 11.2个月）^[58]^。分层随机对照研究^[59]^显示，益气养阴解毒方联合一线化疗可延长中位PFS（11.0 vs 6.0个月），并提高1年OS率（50.0% vs 13.9%）。七叶灵方联合EC方案亦可改善PFS（5.70 vs 3.68个月）、中医证候评分及卡氏体能状态评分，但OS差异未达到统计学意义（17.3 vs 15.7个月）^[60]^。中药联合靶向治疗的证据也相对有限。消岩汤联合安罗替尼维持治疗仅轻度延长复发后PFS（8.7 vs 7.9个月），OS间差异无统计学意义（12.3 vs 11.7个月）^[61]^。

总体而言，中医药作为联合治疗的辅助策略在ES-SCLC的全程管理中具有一定应用价值，可能主要体现在症状改善、毒性管理、生活质量维护及部分患者生存获益等方面。但现有研究普遍存在样本量小、单中心设计及研究方案异质性较大等局限。未来应在规范辨证及干预方案的基础上开展多中心、大样本随机对照研究，统一疗效终点和质量控制标准，并进一步明确中医药的优势人群、最佳介入时机及主要获益维度，推动其由经验性辅助治疗向规范化、循证化的综合管理模式转变。

## 6 ES-SCLC精准治疗的未来发展方向与挑战

含铂依托泊苷化疗联合PD-1/PD-L1抑制剂已确立为ES-SCLC的一线标准治疗模式，以抗血管生成治疗、维持强化及DLL3等肿瘤表面抗原靶向治疗为代表的新型全身治疗策略不断丰富治疗选择，而局部放疗及中医药辅助治疗进一步完善了ES-SCLC全程综合管理体系。然而，ES-SCLC OS获益仍然有限，复发及获得性耐药依旧是影响长期预后的核心问题，联合治疗仍将是ES-SCLC精准治疗发展的重要方向。但联合治疗并不必然带来协同获益，药物相互作用、脱靶效应、毒性叠加及耐药机制复杂化均可能限制其临床转化。未来应进一步明确不同方案在一线诱导、维持、复发进展及局部强化阶段的最佳应用场景，并完善耐药机制研究和毒性管理策略。SCLC高度异质性及谱系可塑性，是精准治疗转化受限的重要原因。目前，PD-L1表达水平、肿瘤突变负荷（tumor mutational burden, TMB）、SLFN11等指标的预测价值尚存在争议。基于ASCL1、NEUROD1、POU2F3及炎症相关特征的分子分型研究，可能为DLL3、B7-H3及Trop-2等新型靶向治疗提供更精准的人群筛选策略。未来需进一步整合基因组、转录组、蛋白组、单细胞测序及肿瘤微环境信息，建立动态分层体系。

近年来，多项中国原创研究推动了ES-SCLC治疗格局的发展。今后应立足我国真实世界人群和医疗资源现状，并整合分子分型、系统治疗、局部治疗、支持治疗和中西医协同管理，形成符合我国国情的ES-SCLC全程管理模式。
